# Exploration of the mechanism of Qi-Xian decoction in asthmatic mice using metabolomics combined with network pharmacology

**DOI:** 10.3389/fmolb.2023.1263962

**Published:** 2023-12-13

**Authors:** Yuhua Lin, Yue Wu, Fuqi Ma, Cuiting Shan, Jialu Ma, Wenguan Li, Huayang Pan, Xiayi Miao, Jinjin Liu, Xiongbiao Wang, Zhenhua Ni

**Affiliations:** ^1^ Department of Respiratory Medicine, Putuo Hospital, Shanghai University of Traditional Chinese Medicine, Shanghai, China; ^2^ Central Lab, Putuo Hospital, Shanghai University of Traditional Chinese Medicine, Shanghai, China

**Keywords:** Qi-Xian decoction, asthma, metabolomics, network pharmacology, palmitic acid

## Abstract

**Introduction:** Qi-Xian Decoction (QXD), a traditional Chinese medicine (TCM) formula consisting of eight herbs, has been clinically used to treat asthma. However, the underlying mechanisms have not been completely elucidated. This study aimed to combine metabolomics and network pharmacology to reveal the mechanism of action of QXD in asthma treatment.

**Methods:** An ovalbumin (OVA)-induced asthma mouse model was constructed to evaluate the therapeutic effects of QXD. Serum metabolomics and network pharmacology were combined to study the mechanism of anti-asthma action as well as the potential target, and related biological functions were validated.

**Results:** The QXD treatment has demonstrated significant protective effects in OVA-induced asthmatic mice, as evidenced by its ability to inhibit inflammation, IgE, mucus overproduction, and airway hyperreactivity (AHR). Metabolomic analysis has revealed a total of 140 differential metabolites associated with QXD treatment. In addition, network pharmacology has identified 126 genes that are linked to the effects of QXD, including TNF, IL-6, IL1β, STAT3, MMP9, EGFR, JUN, CCL2, TLR4, MAPK3 and MAPK8. Through comprehensive gene-metabolite interaction network analysis, seven key metabolites have been identified and associated with the potential anti-asthmatic effect of QXD, with palmitic acid (PA) being the most notable among them. *In vitro* validation studies have confirmed the gene-metabolite interaction involving PA, IL-6, and MAPK8. Furthermore, our research has demonstrated that QXD treatment can effectively inhibit PA-promoted IL-6 expression in MH-S cells and reduce PA concentration in OVA-induced asthmatic mice.

**Conclusion:** The regulation of metabolic pathways by QXD was found to be associated with its anti-asthmatic action, which provides insight into the mechanism of QXD in treating asthma.

## 1 Introduction

Asthma is a heterologous disease influenced by complex interactions between multiple environmental exposures, metabolism, and host immunoregulatory processes. Globally, the total number of people with asthma is estimated to be as high as 334 million (Masoli et al., 2004). Asthma kills approximately 250,000 people worldwide every year and has become a heavy burden on society and families (Ferkol and Schraufnagel, 2014). The pathogenesis, physiopathology, and clinical manifestations of asthma are complex and diverse. Current treatment is mainly based on hormone inhalation and bronchodilators, which are often insufficient in effectively controlling the clinical symptoms of asthma. Traditional Chinese medicine (TCM) has a long history of asthma treatment and good clinical efficacy. However, due to the multiple components of TCM, the anti-asthma mechanism of TCM is often complicated, which limits its wide application.

Metabolomics has been utilized for the quantitative and qualitative analysis of the relative relationship between metabolites and physiological and pathological changes. In recent years, metabolomics has played a crucial role in elucidating possible mechanisms by which TCM treats diseases such as cancer and diabetes (Long et al., 2020; Li et al., 2023). In asthma, several metabolites have been identified to affect airway function and respiratory inflammation (Xu et al., 2021; Yip et al., 2021). For example, cytosolic phospholipase A2 and 15-lipoxygenase metabolites play a significant role in the development of airway hyperresponsiveness (AHR) and airway inflammation (Choi et al., 2005; Jeon et al., 2009). However, metabolomics research is limited as it only presents underlying metabolites and associated pathways without further exploring the relationship between the diseases; thus, data analysis and interpretation are urgently required (Tian et al., 2022). Therefore, the identification of differential metabolites and the revelation of their role in asthma pathogenesis aer crucial. Several studies have demonstrated that network pharmacology provides a new method and concept to study the mechanisms of certain ingredients using computational pharmacology based on existing study results. Based on proteomics, systems biology, pharmacology, and disease pathogenesis, the drug-gene-disease relationship was established using network pharmacology. Therefore, combining metabolomics technology and network pharmacology has advantages in determining the comprehensive mechanism of TCM treatment for asthma.

**FIGURE 1:**
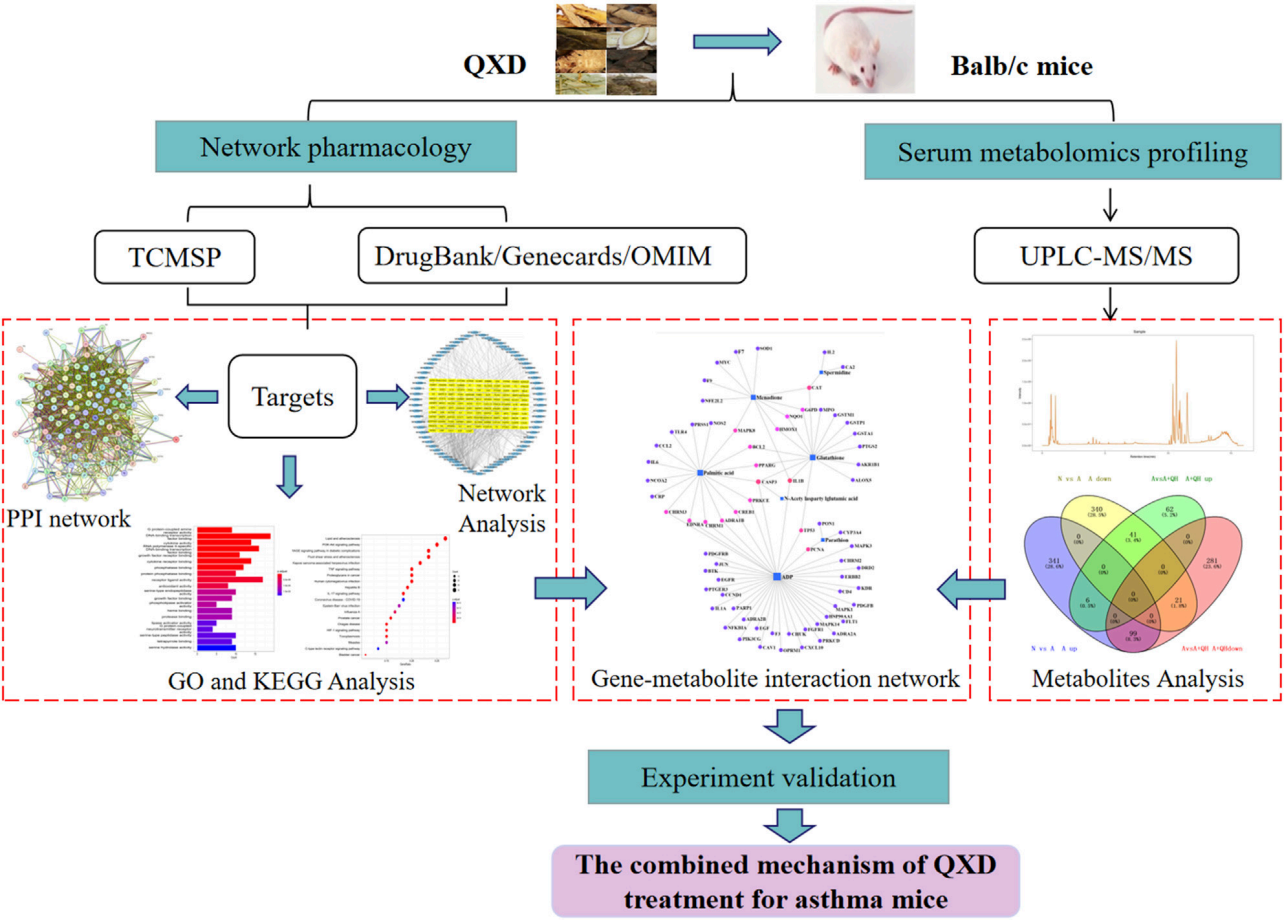
Entire study design based on metabolomics, network pharmacology and experimental verification.

The Qi-Xian Decoction (QXD) has been used in the treatment of asthma for a decade and is composed of eight herbs: *Astragalus membranaceus* (Huangqi), *Epimedium brevicornum* (Yinyanghuo), *Morinda officinalis* (Bajitian), *Polygonum cuspidatum* (Huzhang), *Ligusticum chuanxiong* (Chuanxiong), *Rehmannia glutinosa* (Dihuang), Eriobotrya j *aponica* (Pipaye), *Inula japonica* (Xuanfuhua). Our clinical study has demonstrated that QXD granules (Qixian Qingming Granules) exhibit significant anti-asthmatic effects, such as a notable reduction in exhaled nitric oxide levels and a decrease in the number of acute attacks within 1 year (Bi et al., 2020a). Furthermore, our studies have shown that QXD can alleviate airway inflammation and upregulate E-cadherin expression in human lung epithelial cells and ovalbumin-challenged mice by inhibiting ROS-mediated extracellular signal-regulated kinase (ERK) activation (Tang et al., 2020). However, the anti-asthma mechanism of QXD has not been fully elucidated. To further clarify the effective components, related targets, and pathways of QXD in treating asthma, we conducted metabolomic examination and network pharmacology analysis to identify the possible mechanisms and pathways for QXD in asthma treatment. The basic process of network pharmacological research and metabonomics validation of QXD treatment for asthma (Figure 1).

## 2 Materials and methods

### 2.1 OVA-challenged mouse model of asthma and QXD treatment

Female BALB/c mice were used to build asthmatic model based on Henderson’s study (Henderson et al., 1996). 28 female Balb/c mice (20 ± 2 g), Four-week-old, SPF-grade, were purchased from Shanghai Jihui Experimental Animal Breeding Co., Ltd. License number (SCXK (Shanghai) 2017–0,012), and purchased in the animal room of Putuo District Central Hospital, license number (SYXK (Shanghai) 2022–0,002). The research was conducted in strict accordance with the National Institutes of Health Guide for the Care and received the approval of the institutional and local committees. Mice were randomly divided into four groups (n = 7): normal (N), asthma model (A), QXD low-dose treated (A + QL), and QXD high-dose treated (A + QH) groups. Except for the N group, the mice in the other groups were sensitised sensitizedvia intraperitoneal (ip.) injection of 200 µL sensitizing solution consisting of aluminium hydroxide and OVA on the 1st and 15th days, and then challenged by intranasal administration of 50 μL OVA on days 15, 26, 27, and 28. The mice in the A + QL and A + QH groups were orally administered QXD 0.25 mL and 0.5 mL (0.5 mL equal to a dose of 37.5 g/kg/d (Tang et al., 2020; Zhang et al., 2020) for 14 consecutive days, and the mice in the N and A groups were given 0.9% NaCl. On the 29th day, the mice were anaesthetised with isoflurane, and serum samples were collected for enzyme-linked immunosorbent assay (ELISA) and serum metabolomic analysis, which was performed using ultraperformance liquid chromatography-tandem mass spectrometry (UPLC-MS/MS). Lung tissues were collected for further analysis.

### 2.2 AHR examination

AHR in OVA-challenged mice was assessed using the WBP whole body plethysmography system (Tawang Intelligent, Shanghai, China). Each mouse was exposed to aerosolized doses of either PBS or methacholine (Mch) at concentrations of 3.125, 6.25, and 12.5 mg/mL for a duration of 3 min. Following the exposure, respiratory parameters were measured for a period of 5 min. The enhanced pause (Penh) values were recorded for each mouse in the different experimental groups and subsequently utilized for further analysis (Nilsson et al., 2022).

### 2.3 Cell culture

MH-S murine alveolar macrophage cell line was purchased from Zhong Qiao Xin Zhou Biotechnology Co., Ltd (Shanghai, China). MH-S cells were cultured in Roswell Park Memorial Institute (RPMI)-1,640 medium (Thermo Fisher Scientific, Waltham, United States) supplemented with 0.1% β-mercaptoethanol (Zhong Qiao Xin Zhou Biotechnology Co., Ltd., China), 10% foetal bovine serum (Moregate, Australia), and a penicillin/streptomycin solution. Cells were incubated at 37°C with 5% CO2.

### 2.4 Preparation of bovine serum albumin (BSA)-conjugated palmitic acid (PA)

20 mM solution of PA (Sigma, 57–10–3) in 0.1 M NaOH was incubated at 70°C for 30 min, and was dissolved by mixing with 10% fatty acid-free foetal bovine serum in a 70 °C water bath.

### 2.5 QXD and QXD water extract (QXD-W) preparation

Eight herbs were purchased from Putuo Hospital (Shangyao Huayu Pharmaceutical Co., Ltd., Shanghai, China), and subjected to quality control. These herbs meet the required standards and specifications. The preparation methods used for QXD are consistent and follow the standard operating procedures for Chinese medicine preparations, and by meticulously evaluating the color, appearance, odor, and solubility of each batch of QXD, we can maintain quality control, ensuring consistency in therapeutic effectiveness and patient safety. In brief, two sets of QXD components were boiled with 2 L of water and concentrated to create a boiled water extract (QXD, 272 mL). It was stored at 4°C prior to administration to the mice. The composition of the formula are shown in Table 1. The QXD-W was obtained by using the same method described above, where 272 mL of QXD was obtained. It was then further concentrated to 0.45 L. The concentrated decoction was directly freeze-dried, resulting in 95.75 g of freeze-dried powder with an extraction rate of 30.11%. In vitro studies, the powder was dissolved in distilled water and used at a concentration of 80 ug/mL (Tang et al., 2020).

**TABLE 1:** Composition of QXD.

Herb	Latin name	Application (g)
Huangqi	*Astragalus membranaceus*	30
Yinyanghuo	*Epimedium brevicornum*	30
Bajitian	*Morinda officinalis*	30
Huzhang	*Polygonum cuspidatum*	30
Chuanxiong	*Ligusticum chuanxiong*	15
Dihuang	*Rehmannia glutinosa*	30
Pipaye	Eriobotrya j*aponica*	30
Xuanfuhua	*Inula japonica*	9

### 2.6 Haematoxylin and eosin (HE) and alcian blue (AB)-periodic-acid schiff (PAS) staining

Lung tissues were fixed in formalin solution. Following embedding in paraffin, the tissues were cut into 4 µm sections and stained with HE and AB-PAS solution. Images were captured using a microscope (Olympus BX43, Tokyo, Japan). The inflammation score was evaluated based on the infiltration of inflammatory cells around the airway [no inflammation (0 point); a little (1 point); more but not yet connected in a circle (2 points), a ring of inflammatory cells (3 points), infiltration of a large number of inflammatory cells (4 points)], and the percentage of airway goblet cells [no goblet cell coverage (0 point), <25% (1 point), 25%–50% (2 points), 50%–75% (3 points), and >75% (4 points)] (Jiang et al., 2020).

### 2.7 Real-time quantitative polymerase chain reaction (RT-qPCR)

Total RNA was isolated from the cells and lung tissue using TRIzol reagent (TaKaRa, Dalian, China), and first-strand cDNA synthesis was performed using the PrimeScript RT reagent Kit (TaKaRa, Dalian, China). PCRs were performed using specific forward and reverse primers. RT-qPCR was performed using TB Green Master Mix (TaKaRa, Dalian, China). The relative expression levels of the mucin 5AC (MUC5AC) and IL-6 genes were normalised against β-actin and analysed using the 2^−ΔΔCT^ method, as described previously (Ni et al., 2016). Primer sequences used in this study are listed in Table 2.

**TABLE 2:** Sequences of primers.

Primers	Sequence (5’to3′)
MUC5AC (F)	AAT​GGC​GAG​TCT​GTG​CAG​GA
MUC5AC (R)	CAC​CAG​GTG​TGG​CAT​TGT​GA
IL-6 (F)	TCT​ATA​CCA​CTT​CAC​AAG​TCG​GA
IL-6 (R)	GAA​TTG​CCA​TTG​CAC​AAC​TCT​TT
β-actin (F)	GGC​TGT​ATT​CCC​CTC​CAT​CG
β-actin(R)	CCA​GTT​GGT​AAC​AAT​GCC​ATG​T

### 2.8 Western blot (WB)

Cell lysates were prepared using RIPA lysate buffer and subsequently centrifuged. The resulting supernatant was collected, and the protein concentration was determined using the BCA protein quantification kit (Beyotime, Shanghai, China). The total protein samples were separated on a 10% SDS-PAGE gel and subsequently transferred onto a polyvinylidene fluoride (PVDF) membrane (Miliipore, United States). The PVDF membrane was then blocked with 5% BSA for a duration of 2 h. Subsequently, the membrane was washed three times with Tris-buffered saline and Tween 20 (TBST) and incubated overnight at 4°C with primary antibodies against p-JNK (CST, United States) and GAPDH (Abcam, United States). On the following day, the membranes were washed three times with TBST and incubated with an anti-rabbit IgG horseradish peroxidase secondary antibody at room temperature for 2 h. Images were captured using a gel imaging analyzer (ImageQuant LAS, United States). Quantitative analysis was performed using ImageJ software.

### 2.9 Immunofluorescence

The tissue sections were deparaffinised in xylene, rehydrated in graded ethanol, and boiled in citrate buffer for antigen retrieval. Then, the sections were blocked with 5% BSA for 30 min and incubated with antibodies against CD11c antibody (Thermo Fisher Scientific, Waltham, United States) and IL-6 (Thermo Fisher Scientific, United States) at 4°C overnight. Subsequently, the tissue sections were washed three times with phosphate-buffered saline (PBS) and incubated with secondary antibodies with Alexa Fluor 488-conjugated anti-Syrian hamster IgG (Abcam, Cambridge, United States) and Cy3 conjugated anti-rabbit IgG (Abcam, United States). Finally, the sections were washed and stained with 4′,6-diamidino-2-phenylindole (DAPI). Immunofluorescence images were captured using a microscope (Leica DM13000B, Wetzlar, Germany).

### 2.10 Enzyme-linked immunosorbent assay (ELISA)

Total IgE levels in mouse serum were detected using an IgE ELISA assay kit (Lianke, Hangzhou, China), PA levels in mouse serum were detected using a PA ELISA assay kit (Yaji, Shanghai, China), and IL-6 levels in the cell supernatant were determined using an IL-6 ELISA assay kit (ABclonal, Wuhan, China) according to the manufacturer’s instructions.

### 2.11 Bio-Plex cytokine assays

Cell supernatants were analysed in 96-well microplates according to the manufacturer’s instructions; 23 targets were simultaneously quantified: IL-1α, IL-1β, IL-2, IL-3, IL-4, IL-5, IL-6, IL-9, IL-10, IL-12 (p40), IL-12 (p70), IL-13, IL-17, eotaxin, granulocyte colony-stimulating factor (G-CSF), granulocyte-macrophage (GM)-CSF, interferon (IFN)-γ, keratinocytes (KC), monocyte chemoattractant protein-1 (MCP-1), macrophage inflammatory protein-1 (MIP)-1α, MIP-1β, regulated on activation, normal T cell expressed and secreted (RANTES), and tumour necrosis factor (TNF)-α. Each specific reaction was quantified based on the strength of the fluorescence signal, and the content of each well was then pumped into the Bio-Plex 100 System array reader (Bio-Rad Laboratories, Shanghai, China). The obtained data were processed using Bio-Plex Manager software (version 6.1) and compared with the control group to convert the changes into multiple changes.

### 2.12 Network pharmacology analysis

The DrugBank (https://www.drugbank.ca/), Genecards database (https://www.genecards.org/), and Online Mendelian Inheritance in Man (OMIM) database (https://www.omim.org/) were used to search for asthma-related targets using “asthma” as the key word. The active ingredients from QXD and their corresponding targets were used to generate the “QXD-Compound-Target” network. TCM systems pharmacology database and analysis platform (TCMSP) (http://lsp.nwu.edu.cn/tcmsp.php) was used to search for QXD ingredients under the following screening conditions: oral bioavailability (OB)≥ 30% and drug-like (DL) ≥ 0.18, while the 72 compounds contained in the QXD obtained by mass spectrometry (Zhang et al., 2020), were included in the analysis, Compounds that did not contain the corresponding molecular formulas were excluded, and validated compounds were included in conjunction with literature reports. TCMSP was used to screen for the predicted target genes of the active ingredients. The predicted genes were integrated through UniProt (https://www.uniport.org/) and imported into the Search Tool for Retrieval of Interacting Genes/Proteins (STRING) platform (https://string-db.org/) to establish the protein-protein interaction (PPI) network map as target genes. The predicted genes were imported into Cytoscape 3.7.2 software to construct the “QXD-Compound-Target-Disease” network. In order to further analyse the Gene Ontology (GO) function and Kyoto Encyclopedia of Genes and Genomes (KEGG) pathway of the target genes, R software version 3.6.2 and the Bioconductor software package were used to construct the “drug-target-disease” network.

### 2.13 UPLC-QE-orbitrap-MS/MS detection

Serum sample was mixed with 20 μL of 2-chloro-l-phenylalanine (0.6 mg/mL), and 300 μL ice-cold mixture of methanol and acetonitrile (V:V = 2:1) were added. The mixtures were vortexed for 1 min, and the total samples were extracted by ultrasonication for 10 min in an ice-water bath and stored at −20°C for 30 min. After centrifugation at 13,000 rpm at 4°C for 10 min, 200 μL of the supernatant was dried and redissolved in 300 μL methanol-water (V:V = 1:4), the solution was vortexed for 30 s, extracted by ultrasonication for 3 min in an ice-water bat, then placed at −20°C for 2 h, centrifugation at 13,000 rpm at 4°C for 10 min, 150 μL supernatants were filtered with 0.22 μm microfilters and transferred to LC vials. UPLC-QE-Orbitrap-MS/MS analyses were used to examine in both ESI positive and ESI negative ion modes by using an ACQUITY UPLC HSS T3 (1.8 μm, 100*2.1 mm, Waters, Milford, United States) system. The mobile phase consisted of 0.1% formic acid used as mobile phases A and B, and this process was carried out with an elution gradient (0.01s–16s), delivered at 0.35 mL·min^−1^. The injection volume was 2 μL, and column temperature was 45°C. All the samples were kept at 4 °C during the analysis. The metabolite was detected by mass spectrophotometer scanning using a QE-HF mass spectrum detector (Thermo Fisher Scientific, Waltham, United States) from Oe biotech Co., Ltd (Shanghai, China). Full scan mode (100–1,200 m/z) combined with MSE mode for data acquisition was used. Differential metabolites with variable importance in projection (VIP) values >1.0 and *p*-values <0.05 were selected (Jiang et al., 2021).

### 2.14 Metabolites KEGG analysis and construction of gene-metabolite interaction networks

Using MetaboAnalyst 5.0, differential metabolites were imported into the pathway analysis online program. The pathway involved in different metabolites were analysed using KEGG exploration and visualisation functions. Differential metabolites and target genes were imported into the network analysis online program. The interactions between metabolites and genes were analysed using network exploration and visualisation functions.

### 2.15 Statistical analysis

SPSS version 22.0 software and GraphPad Prism 8.0 (GraphPad Software, La Jolla, CA, United States) were used for data analysis and diagrams. The means of two different groups were compared using unpaired Student’s t-test. One-way or two-way analysis of variance (ANOVA) was used to analyse repeated values when comparing more than two variables. The results are expressed as mean ± standard error of the mean (SEM). A value of *p* < 0.05 was considered statistically significant.

## 3 Results

### 3.1 Protective effects of QXD in an OVA-induced asthma mouse model

**FIGURE 2:**
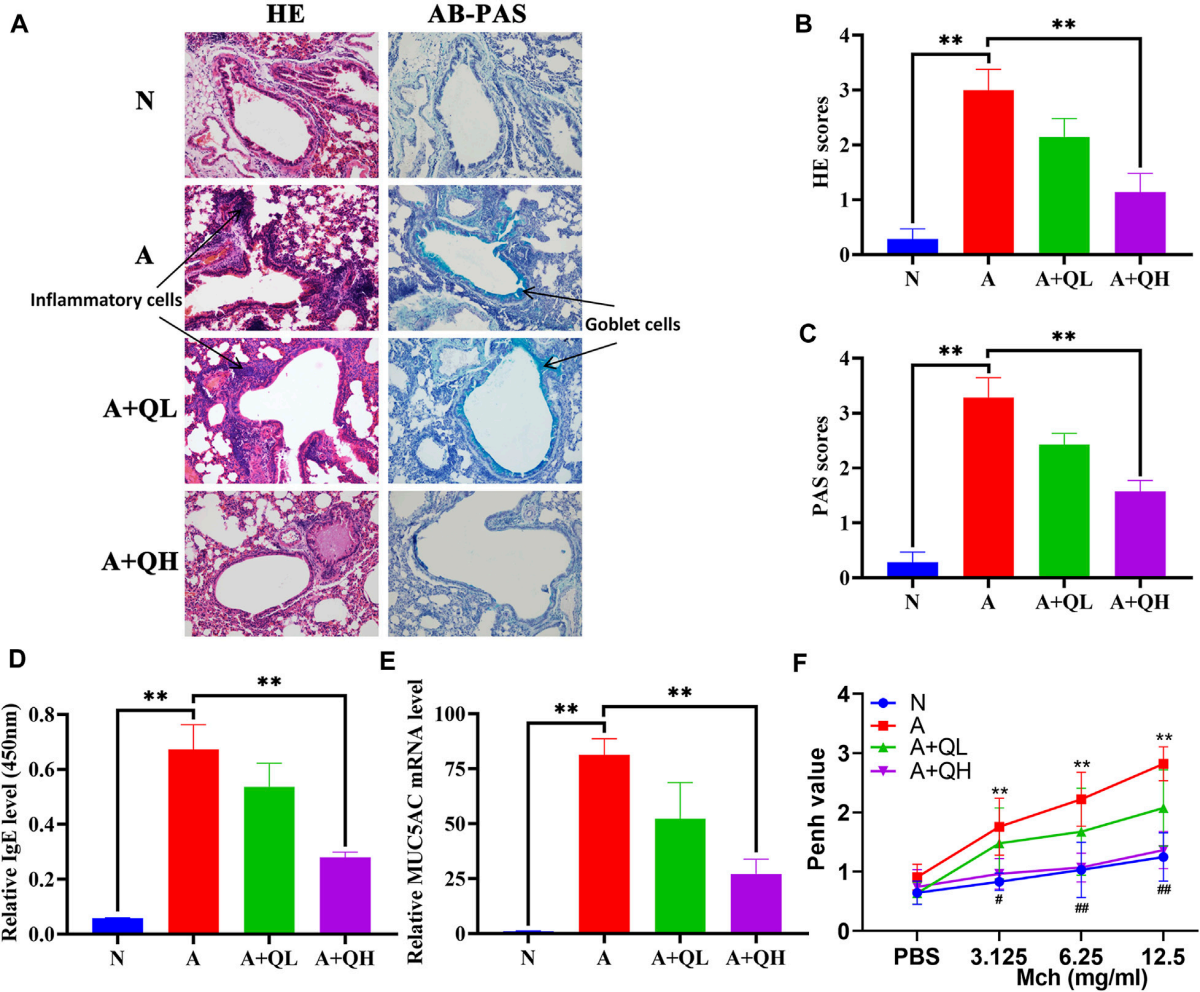
Protective effects of QXD in OVA mice. **(A)** HE and AB-PAS staining of lung tissue compared among control (N), asthma model **(A)**, QXD low-dose treated (A + QL) and QXD high-dose treated (A + QH) groups. Original magnification: ×200 **(B and C)** HE and AB-PAS score statistics chart. **(D)** Serum IgE level was measured using ELISA. **(E)** mRNA expression of MUC5AC in mouse lung was detected with RT-qPCR. **(F)** Pause (Penh) values were detected by nebulized inhalation of Mch at concentrations of 3.125, 6.25 and 12.5 mg/mL, and baseline levels were detected using PBS (N vs. A group:*, A vs. A + QH group:#). Data mean ± S, **p < 0.01, #*p* < 0.05, ##*p* < 0.01, n = 7.

HE staining was used to visualise the alveolar structure and infiltration of inflammatory cells around the airway, while AB-PAS staining was used to determine the distribution of goblet cells and mucus secretion. In comparison to the normal group, the asthma group exhibited increased inflammatory cell infiltration around the airways, smooth muscle hyperplasia, and mucus secretion. After QXD treatment, the A + QH group showed significantly reduced surrounding inflammatory cells and decreased mucus secretion (Figures 2A–C). Serum IgE and MUC5AC expression levels were lower than those in the asthma group (Figures 2D, E). QXD treatment also notably attenuated Penh values in Mch-induced asthma mice (Figure 2F). This results indicates that QXD treatment exerted a protective effect in an OVA-induced asthma mouse model by inhibiting inflammation, mucus overproduction, IgE and AHR.

### 3.2 Serum metabolomics analysis

**FIGURE 3:**
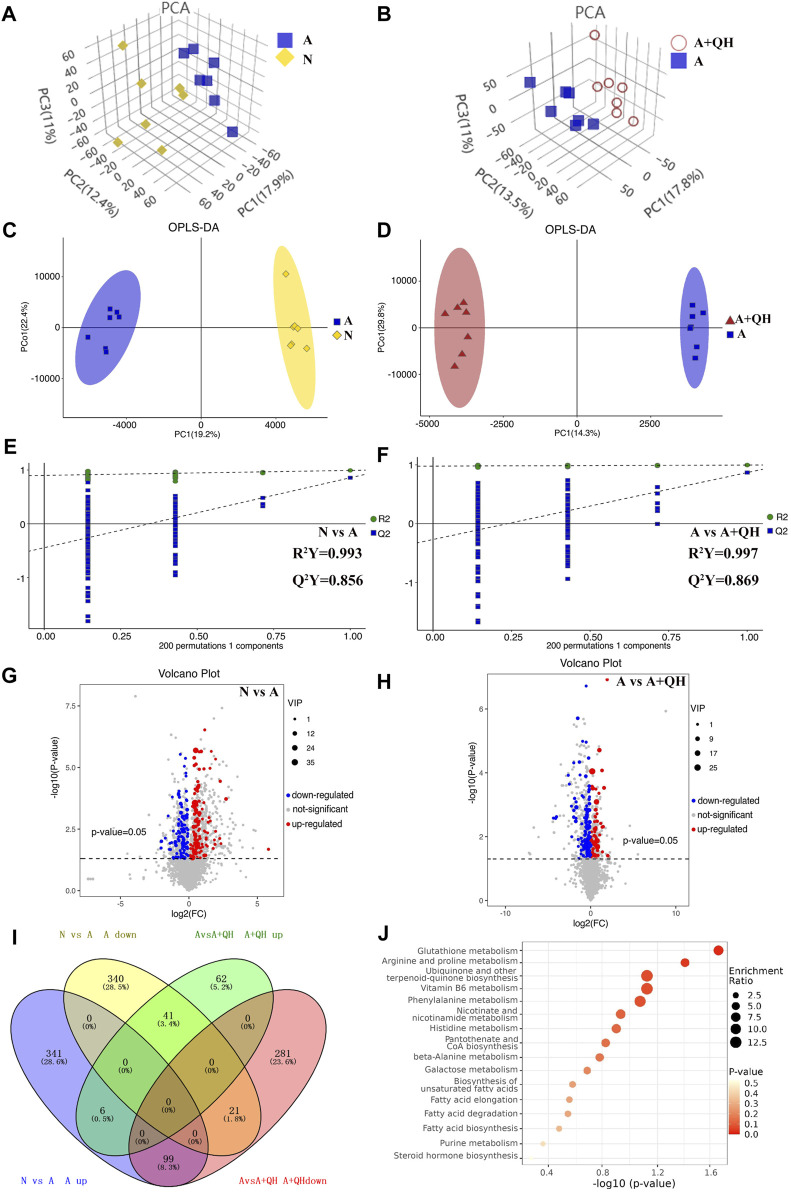
Serum metabolomics profiling. **(A and B)** 3D PCA score plot. **(C and D)** OPLS-DA score plot. **(E and F)** 7-fold cross validation plot of OPLS-DA model with 200 permutation tests. **(G and H)** Volcano plot analysis showing differences in metabolites between the groups, The differential metabolites were selected using the criteria of *p* < 0.05 and variable importance in projection (VIP) value >1. A, C, E and G show the comparison between the N and A groups. B, D, F and H show the comparison between the A and A + QH groups. **(I)** Venn diagram analysin g differential metabolites between the N, A, and A + QH groups, The differential metabolites were selected based on the ratio of detected values and analyzed in conjunction with a significance threshold of *p* < 0.05. **(J)** Metabolic pathways were analysed based on 140 different metabolites by MetaboAnalyst 5.0, n = 7.

Based on a 7-fold cross-validation, unsupervised principal component analysis (PCA) was employed for dimensionality reduction. The tight clustering of samples within the same group indicated the overall distribution and stability of the entire analysis process (Figures 3A, B). Subsequently, a supervised orthogonal partial least squares discriminant analysis (OPLS-DA) score plot showed that an obvious separation between the samples of the N group and the A group (Figure 3C), and the A + QH group was obvious separation between the samples of the A group (Figure 3D), suggesting that biochemical changes differed between the groups. As showed in Figures 3E, F, R2Y = 0.993 (N vs. A), Q2Y = 0.856 (N vs. A), R2Y = 0.997 (A vs. A + QH), Q2Y = 0.869 (A vs. A + QH), indicated that the N, A and A + QH groups were not overfitted. Using untargeted metabolomics, a total of 13,420 substance peaks were detected, including 4,417 metabolites. Among them, there are 2,461 anionic metabolites and 1,956 cationic metabolites (Supplementary Table S1). Moreover, we visualised the significantly upregulated (red dots) and downregulated (blue dots) metabolites, which were screened for differential metabolites in the form of volcano plots. Using intergroup comparison method, compared to the N group, 118 metabolites were upregulated and 102 were downregulated in the A group (Figure 3G). Compared to the A group, 59 metabolites were upregulated and 139 metabolites were downregulated in the A + QH group (Figure 3H). The top 30 metabolites with significant changes are shown in Table 3. We performed a combined analysis of differential metabolites in the three groups using Venn online software and found that 99 metabolites were increased in the A group, which were downregulated after QXD treatment, and 41 metabolites were decreased in the A group, which increased after QXD treatment (Figure 3I). Related differential metabolites and metabolic pathways are presented in (Supplementary Table S2). These 140 differential metabolites may be the key metabolites in QXD treatment of asthma. We further analysed the pathways of these 140 metabolites and showed the top 16 representative pathways (Figure 3J, *p* < 0.05). From the metabolites KEGG analysis, top KEGG terms were related to the glutathione metabolism, arginine and proline metabolism, ubiquinone and other terpenoid-quinone biosynthesis, Phenylalanine metabolism, Biosynthesis of unsaturated fatty acids, Fatty acid elongation, Fatty acid degradation and Fatty acid biosynthesis metabolism signalling pathway.

**TABLE 3:** Differential metabolic signature after QXD treatment of asthma.

NO	Metabolites	Formula	M/Z	RT (min)	VIP	Log2 (A/N)	Log2 (AQH/A)
1	7 (14)-Bisabolene-2,3,10,11-tetrol	C15H28O4	317.1973406	5.691566667	0.30	3.54↑**	−1.19↓*
2	Callystatin A	C29H44O4	501.3229614	9.04485	1.10	3.27↑**	−2.21↓*
3	Citalopram (propionic acid derivative)	C19H16FNO3	649.2146311	5.426183333	0.53	3.11↑**	−0.95↓*
4	Palmitic acid	C16H32O2	301.2388799	9.61905	0.36	3.08↑*	−1.83↓*
5	Uzarigenin 3-[xylosyl-(1->2)-rhamnoside]	C34H52O12	670.379459	7.641516667	0.72	2.90↑**	−0.82↓*
6	Methyl (R)-9-hydroxy-10-undecene-5,7-diynoate glucoside	C18H24O8	413.1458462	4.9061	0.38	2.89↑**	−0.89↓*
7	Pisumionoside	C19H32O9	403.1977175	4.765166667	0.42	2.60↑**	−3.60↓**
8	Cinncassiol C	C20H28O7	425.1823674	7.638033333	0.52	2.49↑**	−1.43↓**
9	RHODOMYRTOXIN B	C24H28O7	473.1822158	6.938133333	0.55	2.42↑**	−1.01↓**
10	(2E,6E)-1-Hydroxy-2,6,10-farnesatrien-9-one	C15H24O2	471.3485522	11.44598333	0.83	2.29↑**	−2.08↓**
11	MG (0:0/14:1 (9Z)/0:0)	C17H32O4	345.2286739	6.818266667	0.45	2.20↑**	−0.88↓*
12	17-phenyl trinor PGF2α isopropyl ester	C26H38O5	475.2683473	7.916783333	0.60	2.18↑**	−1.95↓*
13	(±)-1,4-Nonanediol diacetate	C13H24O4	289.166026	5.013016667	0.35	2.16↑**	−1.02↓*
14	QX-314	C16H26N2O	307.2031347	9.206716667	0.47	1.97↑**	−0.89↓*
15	3alpha,4beta,7alpha-Trihydroxy-5beta-cholan-24-oic Acid	C24H40O5	453.2864381	7.916783333	1.46	1.95↑**	−1.46↓*
16	Pantetheine	C11H22N2O4S	279.1376638	4.5677	0.19	−4.49↓*	5.54↑*
17	Lubiminol	C15H26O3	299.1867329	9.935016667	0.34	−3.58↓*	4.60↑**
18	20-Oxo-leukotriene E4	C23H35NO6S	452.2101721	7.251733333	0.53	−2.12↓**	2.26↑*
19	2-Naphthalenesulfonic acid	C10H8O3S	189.0021432	4.574983333	1.02	−2.08↓*	2.16↑*
20	N-(3-oxo-dodecanoyl)-homoserine thiolactone	C16H27NO3S	358.1699534	8.896166667	0.68	−1.87↓**	2.22↑*
21	Spermidine	C7H19N3	146.1651725	0.566383333	0.35	−1.82↓*	1.24↑*
22	DOPA sulfate	C9H11NO7S	322.0247113	4.5254	0.25	−1.62↓**	0.99↑*
23	17-dimethylarsinoyl-9Z-heptadecenoic acid	C19H37AsO3	387.1889931	3.607783333	0.15	−1.57↓**	1.27↑**
24	N- (3-TRIFLUOROMETHYLPHENYL)PIPERAZINE (TFMPP)	C11H13F3N2	459.2006528	7.251733333	0.49	−1.44↓*	1.35↑*
25	Meptazinol glucuronide	C21H31NO7	408.2032591	7.2661	1.38	−1.39↓*	1.39↑*
26	2-(2H-1,3-benzodioxol-5-yl)-2-oxoacetic acid	C9H6O5	232.9854875	4.5677	0.95	−1.38↓*	1.83↑*
27	4-Chloro-17alpha-methyl-17beta-hydroxy-4-androsten-3-one	C20H29ClO2	381.1842397	7.251733333	0.88	−1.34↓*	1.23↑*
28	Trifluridine	C10H11F3N2O5	277.0442351	4.5254	0.48	−1.25↓*	0.67↑*
29	N-Acetylaspartylglutamic acid	C11H16N2O8	303.0836921	1.1402	0.60	−1.12↓**	0.64↑**
30	DEQUALINIUM	C30H38N4	472.3427942	10.25435	0.58	−1.09↓**	0.70↑*

↑ indicates increase; ↓ indicates decrease. *p < 0.05; * *p < 0.01.

### 3.3 Network pharmacology analysis

**FIGURE 4:**
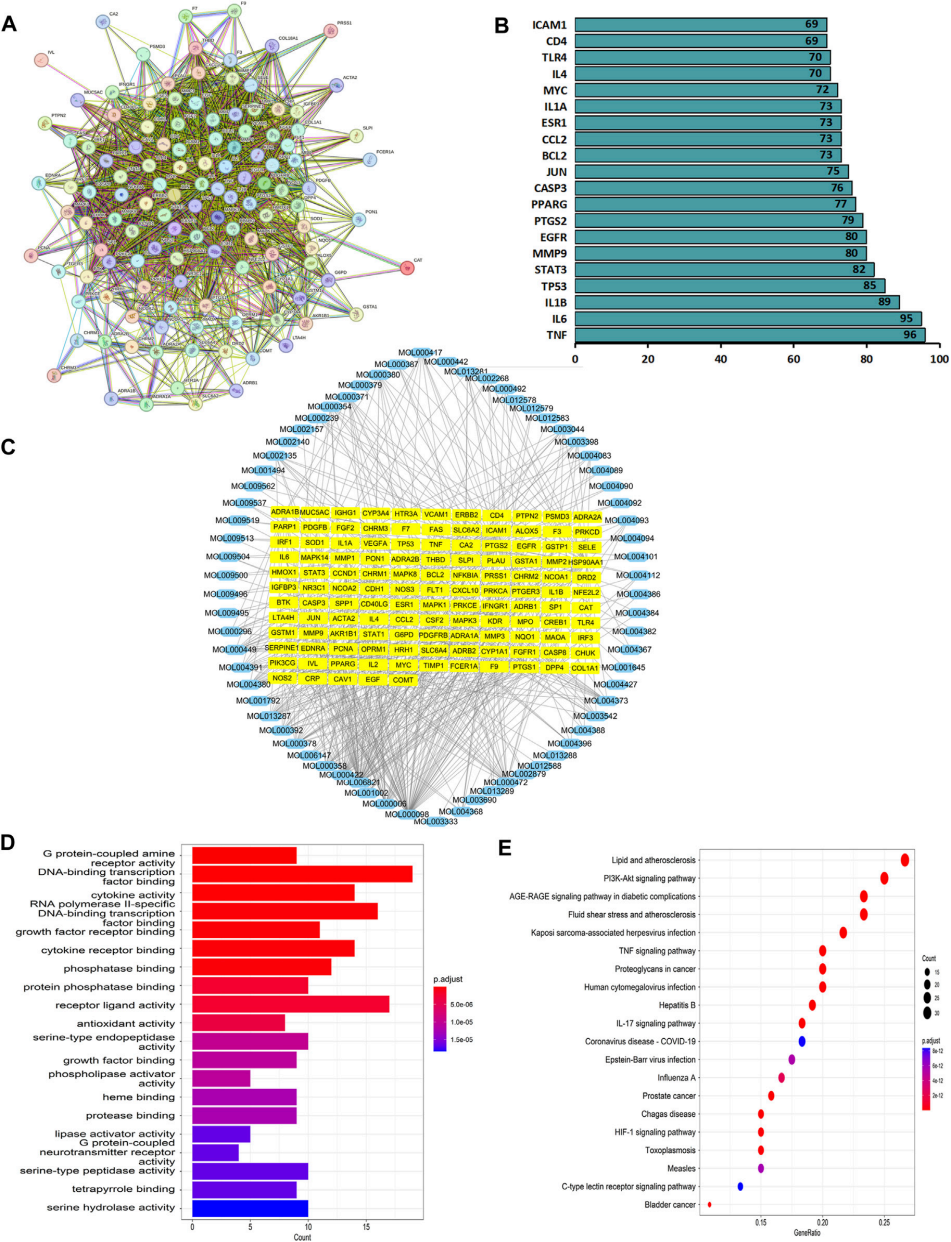
Network pharmacology analysis. Based on the 126 target genes **(A)** PPI network analysis. **(B)** Connection node count of PPI network. **(C)** Cytoscape software were used to analyse the relationship between 126 genes and 69 compounds. **(D)** GO functions terms analysis. **(E)** KEGG pathways analysis.

Network pharmacology was used to construct the asthma-gene-QXD network. We identified asthma-related genes (1,091 gene symbols) and core targets (2,448 gene symbols) corresponding to compounds contained in 8 herbs. We screened 126 genes associated with asthma and QXD (Table 4). Based on these 126 genes, we conducted protein-protein interaction (PPI) network analysis to display the interactions between these genes (Figure 4A). The top 20 genes are depicted in Figure 4B, which can be considered as core targets in the PPI network. Cytoscape was used to map the node relationships between 126 genes and 69 compounds (Figure 4C). After enrichment analysis of the GO function terms and KEGG pathways, 132 GO terms and 164 KEGG pathways were obtained. According to the number of enriched genes and P. adjust value <0.05, we selected the top 20 GO terms (Figure 4D) and KEGG pathways (Figure 4E). In the GO function analysis, the top GO terms associated with asthma were G protein-coupled amine receptor activity, cytokine activity, growth factor receptor binding, cytokine receptor binding, phosphatase binding, receptor ligand activity, antioxidant activity, growth factor binding, and MAP kinase activity. From the KEGG enrichment analysis, the top KEGG terms were related to Lipid and atherosclerosis, TNF signaling pathway, PI3K-Akt signaling pathway, HIF-1 signaling pathway, Toll-like receptor signaling pathway, MAPK signalling pathway and EGFR tyrosine kinase inhibitor resistance. These GO functions and KEGG pathways were closely associated with cytokines, growth factors, antioxidants, and inflammation; detailed genes and pathways are shown in (Supplementary Table S3).

**TABLE 4:** Gene associated with both asthma and QXD.

Gene Symbol (126 genes)
ADRB2, CHRM3, PTGS1, PTGS2, HSP90AA1, CHRM1, ADRA1A, CHRM2, ADRA1B, SLC6A4, OPRM1, BCL2, JUN, CASP3, CASP8, PRKCA, PON1, ESR1, DPP4, NOS2, PPARG, KDR, MAPK14, NR3C1, ADRA2A, SLC6A2, AKR1B1, PLAU, LTA4H, MAOA, ADRB1, HTR3A, IL4, MAPK8, MMP1, STAT1, HMOX1, CYP3A4, CYP1A1, ICAM1, SELE, VCAM1, ALOX5, GSTP1, PSMD3, GSTM1, SLPI, MMP3, EGFR, VEGFA, CCND1, MMP2, MMP9, MAPK1, EGF, IL6, NFKBIA, SOD1, ERBB2, CAV1, F3, IL1B, CCL2, PTGER3, NOS3, IL2, THBD, SERPINE1, COL1A1, IFNGR1, IL1A, MPO, NFE2L2, NQO1, PARP1, CRP, CXCL10, CHUK, SPP1, IGFBP3, CD40LG, IRF1, CA2, ADRA2B, CAT, PCNA, GSTA1, IRF3, STAT3, FGFR1, FLT1, PDGFB, MAPK3, PDGFRB, IVL, COMT, TIMP1, EDNRA, CD4, FGF2, FAS, FCER1A, TLR4, SP1, G6PD, CREB1, CDH1, MUC5AC, HRH1, DRD2, ACTA2, BTK, CSF2, IGHG1, F7, F9, MYC, NCOA1, NCOA2, PRKCD, PRKCE, PRSS1, PTPN2, TNF, TP53, PIK3CG

### 3.4 Gene-metabolite interaction network with hub genes and metabolites

**FIGURE 5:**
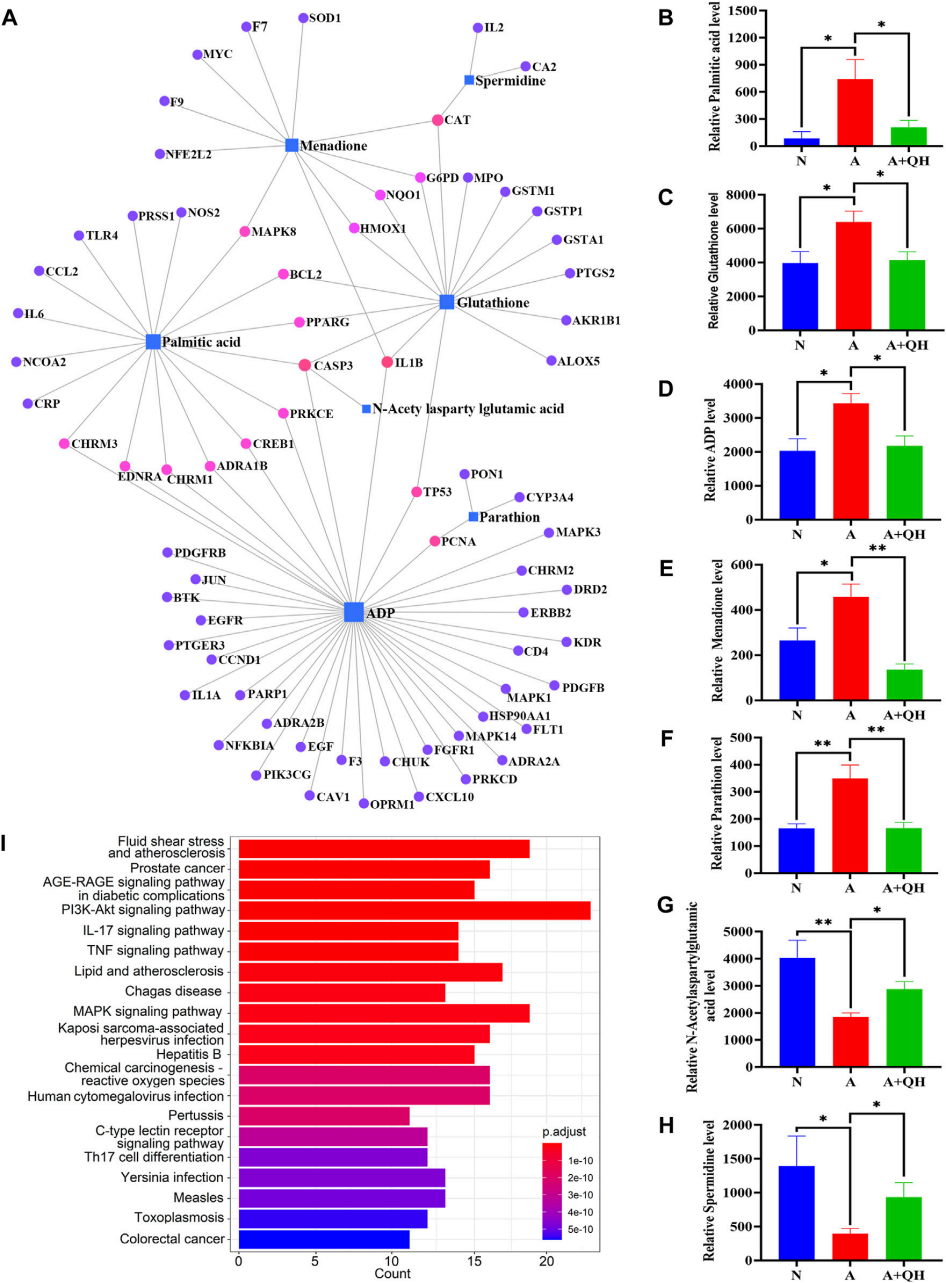
Gene-metabolite interaction network with hub genes and metabolites. **(A)** MetaboAnalyst was used to analyse the interactions of 126 target genes with 140 differential metabolites. **(B–H)** The expression level of 7 differential metabolites were examined by UPLC-MS/MS detection. Relative peak area of differential metabolites in the serum of mice in each group was plotted using GraphPad Prism 8 software. **(I)** KEGG pathways analysis based on the 71 target genes included in Figure 5A. Data mean ± S, *p < 0.05, **p < 0.01, n = 7.

We further elucidated the regulatory effects of these differential metabolites on the key genes involved in asthma pathogenesis by analyzing the gene-metabolite interaction network using MetaboAnalyst 5.0 to explore the interactions between target genes and serum metabolites (Figure 5A). The target genes and related compounds corresponding to the relevant differential metabolites are listed in (Supplementary Table S4). Notably, a total of 7 hub metabolites, namely, PA, glutathione, adenosine diphosphate (ADP), menadione, parathion, N-acetylaspartylglutamic acid, and spermidine, were identified. These metabolites exhibited distinct responsiveness to QXD treatment, as showed in Figures 5B–H. Subsequently, the 71 genes depicted in Figure 5A underwent KEGG analysis, leading to the identification of the top 20 signaling pathways (Figure 5I). Within this subset of pathways, it became evident that PA emerged as the metabolite involved in the most pathways, thereby signifying the pivotal and potiential role of PA in mediating the effect of QXD. Furthermore, to validate our findings, the levels of PA in mouse serum were assessed using ELISA, yielding results that consistently mirrored the trends observed in the mass spectrometry findings (Supplementary Table S5). Collectively, our results strongly suggest that QXD may regulate the concentration of metabolites to exert an anti-asthmatic effect.

### 3.5 QXD inhibits PA-induced IL-6 expression in MH-S cells

**FIGURE 6:**
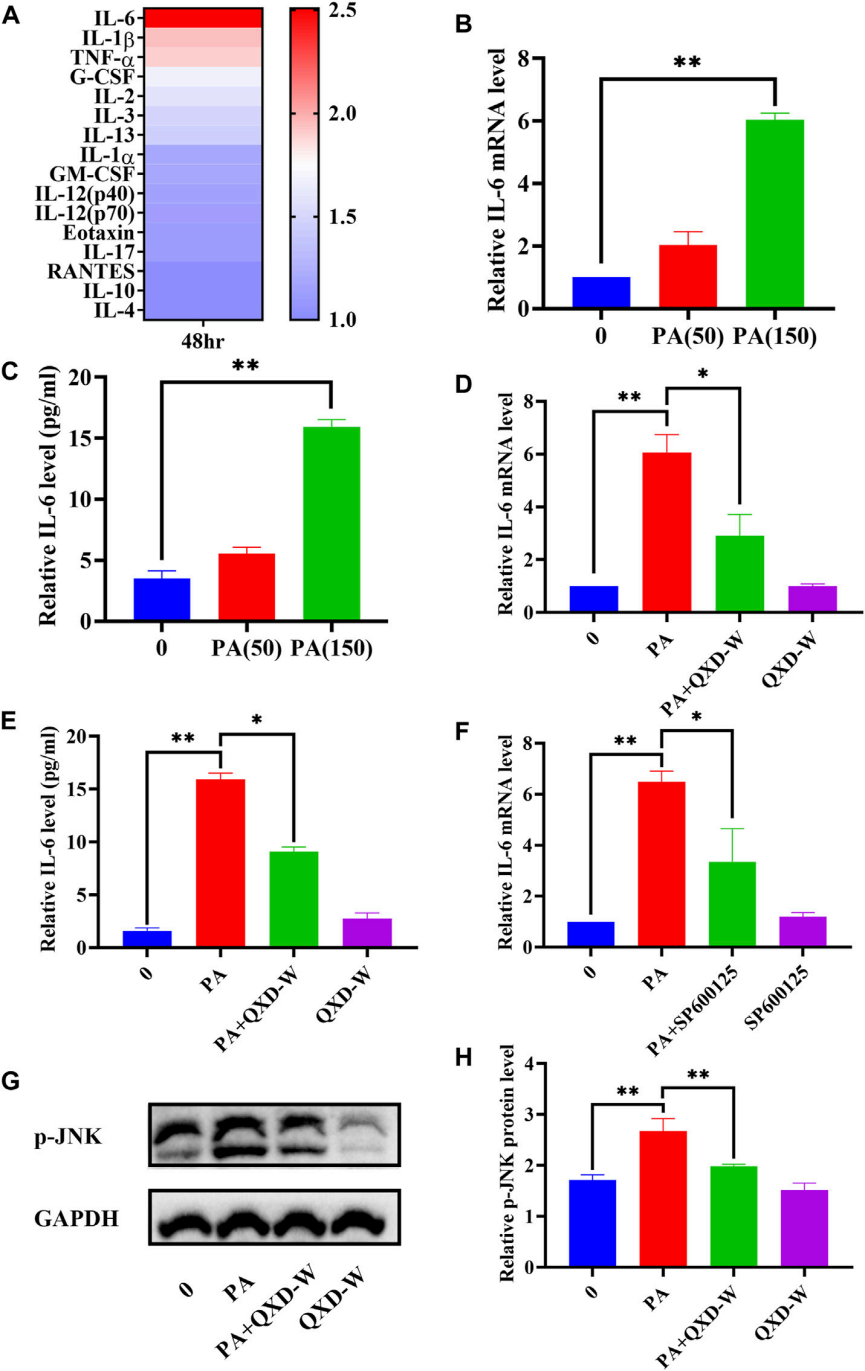
QXD inhibits PA-induced IL-6 expression in MH-S cells. **(A)** MH-S cells were treated with PA (150 μm) for 48 h and the levels of 23 cytokines in the cell supernatants were measured by Bio-Plex Assays. **(B)** MH-S cells were treated with PA (50 μm and 150 μm) for 24 h and IL-6 mRNA expression levels were examined with RT-qPCR. **(C)** MH-S cells were treated with PA (50 μm and 150 μm) for 48 h, and IL-6 secretion were examined using ELISA. **(D)** MH-S cells were treated with PA (150 μm) alone or in conjunction with QXD-W (80 μg/mL) for 24 h, and IL-6 mRNA expression levels were examined with RT-qPCR. **(E)** MH-S cells were treated with PA (150 μm) alone or in conjunction with QXD-W (80 μg/mL) for 48 h, and IL-6 secretion were examined using ELISA. **(F)** MH-S cells were treated with PA (150 μm) and SP600125 (A JNK inhibitor, 25 μm) alone or in combination for 24 h, and IL-6 mRNA expression levels were examined with RT-qPCR. **(G)** PA (150 μm) and QXD-W (80ug/mL) alone or in combination were used to intervene MH-S cells for 6 h, p-JNK protein expression levels were detected by WB. **(H)** ImageJ software was used to analyze the phosphorylation levels of p-JNK proteins. Data mean ± S, *p < 0.05, **p < 0.01, n = 3.

A previous study indicated that lung macrophages are important target cells of PA (Tashiro et al., 2017). In this study, we utilized MH-S lung macrophages to investigate the impact of PA on cytokine secretion. As depicted in Figure 6A, PA treatment exhibited a significant increase in the secretion of IL-6, IL-1β, and TNF-α by MH-S cells. Through gene-metabolite interaction and network pharmacology analysis, we identified a potential association between PA, IL-6, and MAPK8 (JNK), which could potentially be linked to the anti-asthmatic effect of QXD. To validate this hypothesis, we examined the expression of IL-6 mRNA (Figure 6B) and its secretion (Figure 6C) upon PA treatment. Moreover, we observed that the JNK inhibitor SP600125 effectively inhibited the PA-induced expression of IL-6 (Figure 6F). Notably, treatment with QXD-W was able to reverse the PA-induced expression and secretion of IL-6 (Figures 6D, E), as well as the phosphorylation level of MAPK8 (JNK) protein in MH-S cells (Figures 6G, H). These findings provide compelling evidence that PA treatment can stimulate IL-6 production in MH-S cells, and QXD-W may counteract the PA-induced expression of IL-6 in MH-S cells by inhibiting the JNK signaling pathway.

### 3.6 The effect of QXD on IL-6 production in an OVA-induced asthma mouse model

**FIGURE 7:**
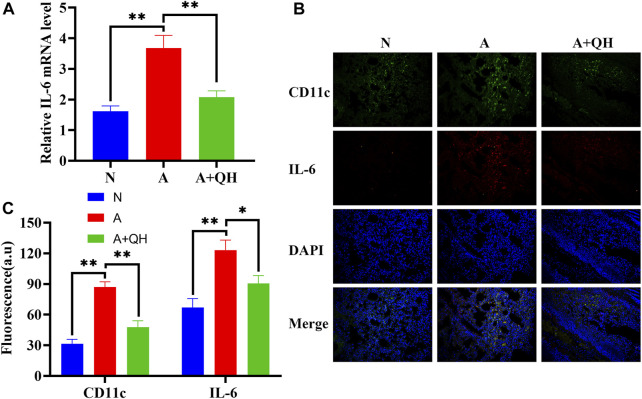
The effect of QXD on IL-6 production in an OVA-induced asthma mouse model. **(A)** IL-6 mRNA expression in mice lung were examined with RT-qPCR. **(B and C)** CD11c and IL-6 expression was detected using immunofluorescence, original magnification: ×200. Data mean ± S, **p < 0.01, n = 7.

To further investigate the therapeutic effects of QXD, we evaluated its impact on IL-6 production in an OVA-induced asthma mouse model. As expected, the relative IL-6 mRNA expression in the A group was significantly higher than that in the N group, whereas QXD treatment significantly downregulated IL-6 mRNA expression (Figure 7A). We also examined the expression of IL-6 in macrophages in lung tissues of OVA mice using immunofluorescence analysis. Our results showed that the increased number of macrophages in the A group was accompanied by a corresponding increase in IL-6 expression in macrophages. However, following QXD treatment, the number of macrophages decreased, and the IL-6 expression in them was also reduced (Figures 7B, C). Taken together, our results demonstrate that QXD treatment effectively decreased the PA concentration and inhibited IL-6 production in lung macrophages in an OVA-induced asthma mouse model.

## 4 Discussion

In this study, we employed metabolomics methods and pharmacology network analysis to elucidate the anti-asthma mechanism of QXD. Serum metabolomics investigation of mice identified 140 QXD-regulated differential metabolites (Figure 3I), with PA being the metabolite involved in the most pathways (Figure 3J). Using network pharmacology, we created the asthma-gene-QXD network to further clarify the regulatory effects of these distinct metabolites on the important genes of asthma pathogenesis. The network pharmacology analysis revealed 126 genes related to the anti-asthmatic effect of QXD, including TNF, IL-6, IL1β, STAT3, MMP9, EGFR, JUN, CCL2, TLR4, MAPK3, and MAPK8 (Table 4). Subsequently, gene-metabolite interaction network analysis using the 126 targeted genes and 140 serum metabolites identified 7 metabolites, which had potential interactions with the core genes of QXD. Among these metabolites, we found that PA closely interacted with key target genes of QXD, including IL-6, CCL2, TLR4, and MAPK8 (Figure 5A). Through UPLC-QE-Orbitrap-MS/MS (Figure 5B) and ELISA (Supplementary S5) testing, it was discovered that QXD can reduce the serum levels of PA in asthmatic mice. Subsequent *in vitro* investigations demonstrated that PA can significantly increase IL-6 production and expression in MH-S, a macrophages cell line. However, QXD-W was able to reverse the expression and secretion of IL-6 induced by PA in MH-S cells (Figures 6D,E). Furthermore, blocking the MAPK8 (JNK) pathway can inhibit PA-induced IL-6 expression in MH-S cells, while QXD-W is capable to inhibiting the JNK signaling pathway (Figures 6F, G). *In vivo* experiments consistently showed that QXD therapy reduced IL-6 expression in lung macrophages in an OVA-induced asthma mouse model (Figure 7). Our results suggest that QXD may decrease airway inflammation in asthmatic mice by reducing blood PA levels and PA-induced macrophage IL-6 production and release.

Based on the gene-metabolite interaction network (Figure 5A), 7 differential metabolites were enriched, including PA, glutathione, ADP, menadione, parathion, N-acetylaspartylglutamic acid, and spermidine. Additionally, according to the 16 representative pathways based on 140 different metabolites (Figure 3J), PA was the metabolite involved in the most pathways, including biosynthesis of unsaturated fatty acids, fatty acid elongation, fatty acid degradation, fatty acid biosynthesis pathway, indicating a close relationship between PA and lipid metabolism. The involvement of PA in inflammation has been previously reported. For instance, Wood et al. demonstrated that neutrophils and monocytes release IL-1β when exposed to a combination of PA and lipopolysaccharides (LPS) or TNF-α. (Wood et al., 2019). Furthermore, studies have shown that PA increases the population of lung macrophages in a mouse model of airway inflammation induced by house dust mite, and directly stimulates these macrophages to produce inflammatory cytokines and chemokines, thereby highlighting the significance of pulmonary macrophages as target cells for PA (Tashiro et al., 2017). In our study, we confirmed that PA treatment significantly enhanced the production of IL-6, IL-1β, and TNF-α in lung macrophages. Furthermore, through gene-metabolite interaction network analysis, we identified a connection between IL-6 and PA. Based on these observations, we hypothesized that the therapeutic effect of QXD in asthma treatment may involve the regulation of PA content and function, potentially achieved through the modulation of macrophage IL-6 expression. To investigate this hypothesis, we conducted experiments using MH-S cells. Our results demonstrated that PA stimulation promoted the secretion of IL-6 by these cells. However, treatment with QXD-W effectively reversed this effect. Additionally, *in vivo* investigations revealed that QXD treatment reduced the expression of IL-6 in lung macrophages of asthmatic mice. Collectively, our findings suggest that PA plays a pro-inflammatory role in asthma, and inhibiting PA levels can potentially alleviate airway inflammation associated with this condition. Moreover, QXD treatment exhibits the ability to suppress PA levels and its associated functions, thereby presenting a potential therapeutic strategy for asthma management.

IL-6 is the most widely active cytokine, and plays an crucial role in airway inflammation. It acts as a significant factor in the differentiation of CD4 T cells into specific effector CD4 cells and modulates the Th1 and Th2 balance (Rincón et al., 1997; Jahreis et al., 2017). According to PPI network analysis, IL-6 contains 95 nodes, and the IL-6 gene in the GO enrichment analysis was primarily enriched in cytokine activity, growth factor receptor binding, cytokine receptor binding, receptor ligand activity, and growth factor activity. KEGG enrichment analysis involved 48 entries, mainly enriched in the AGE-RAGE signaling pathway in diabetic complications, lipid and atherosclerosis, TNF signaling pathway, HIF-1 signalling pathway, MAPK signaling pathway and EGFR tyrosine kinase inhibitor resistance pathway. These results align with current reports indicating that IL-6 and its associated signalling pathways play an crucial role in pulmonary immune system diseases. Various stimuli can induce these cells to produce IL-6, such as smoke, ROS, microbial products, viruses, or other pro-inflammatory cytokines (Lorente et al., 2001; Strzelak et al., 2018) NF-κB, MAPK, toll-like receptor (TLR)4, JNK/ERK, and Akt/mTOR, have been shown to be associated with the expression of IL-6 (Nam et al., 2006; Wang et al., 2022; Yu et al., 2022). Macrophages are pivotal cells in immunity and inflammation, producing IL-6 and IL-1 (Mizel et al., 1989). IL-6 is known to be intricately linked to lipid metabolism, yet the precise mechanism by which metabolites regulate IL-6 secretion in macrophages remains poorly understood. In our study, we made a significant observation that PA treatment significantly enhanced the expression and secretion of IL-6. Importantly, we found that inhibition of the JNK signaling pathway effectively suppressed the PA-induced IL-6 secretion in MH-S cells. Furthermore, our results demonstrated that QXD-W treatment inhibited the phosphorylation of JNK protein, indicating that QXD may exert its inhibitory effects on IL-6 expression and secretion in MH-S cells through the MAPK signaling pathway. This novel mechanism unraveled in our study sheds light on the molecular basis by which QXD modulates macrophage IL-6 expression and secretion. Additionally, our findings suggest that IL-6 could be a pivotal target for the anti-inflammatory properties of QXD, further highlighting its therapeutic potential in combating inflammation.

In addition to PA, six other metabolites were enriched in the gene-metabolite network diagram. Glutathione plays an important role in redox reactions. Patients with asthma often have elevated levels of glutathione in lung samples (Reynaert et al., 2011). ADP is the product of rapid degradation of ATP by extracellular enzymes (mainly CD39 and CD73) and plays an important role in bronchoconstriction, cough, mechanical ventilation-induced lung injury, and idiopathic pulmonary fibrosis (Pelleg et al., 2021). Menadione is a vitamin K3 analogue and potent toxic ROS donor that induces oxidative stress in cells and reduces intracellular glutathione concentration (Tetef et al., 1995; Bertolini et al., 2020). Parathion is positively correlated with wheezing; low levels of parathion activate macrophages to release TNF-α and also affect the production of IgE, Th1, and Th2 cytokines (Hoppin et al., 2006; Fukuyama et al., 2011; Proskocil et al., 2013). N-acetylaspartylglutamic acid can treat allergic diseases, and its level is depleted in allergic diseases (Lai et al., 1989; Preece et al., 1993). Furthermore, we found that the arginine and proline metabolic pathways are other signalling pathways for 140 target metabolites, including spermidine and 4-Guanidinobutanoic acid. Spermidine levels were decreased in the A group compared with those in the N group, and significantly increased after QXD administration. The expression trend of 4-Guanidinobutanoic acid was opposite. Spermidine levels are decreased in bronchoalveolar lavages from asthma patients compared to that in healthy controls (Wawrzyniak et al., 2021). 4-Guanidinobutanoic acid levels are elevated in fibromyalgia, a disease characterised by generalized chronic pain (Hsu et al., 2022). These previous observations are consistent with our findings. Therefore, metabolites such as PA, glutathione, ADP, menadione, parathion, N-acetylaspartylglutamic acid, spermidine, and 4-guanidinobutanoic acid could be considered potential biomarkers for asthma. According to metabolite pathway analysis (Figure 3J), glutathione is involved in glutathione metabolism pathway. ADP is a key metabolite in purine metabolism pathway and also interact with multiple key targets in Figure 5A. We believe that ADP may also play a crucial role in QXD treatment for asthma, and we will conduct further exploration in our next phase of research. Menadione is associated with ubiquinone and other terpenoid-quinone biosynthesis pathway. Spermidine is involved in several metabolic pathways, including glutathione metabolism, Arginine and proline metabolism and beta-Alanine metabolism pathway. Future studies should therefore focus on these signalling pathways. Our report demonstrated that QXD can modulate the roles of these metabolites and pathways in the treatment of asthma. However, the mechanism by which QXD reduces these metabolites requires further investigation.

Network pharmacology has emerged as a powerful approach for comprehensively elucidating the mechanisms underlying the therapeutic effects of traditional Chinese medicine (TCM). In our study, we employed TCM Systems Pharmacology (TCSMP) to analyze the effective compounds present in QXD, combined with the compounds previously identified through mass spectrometry (Zhang et al., 2020). Through this integrative analysis, we successfully identified 69 compounds (Figure 4C) associated with asthma. To further explore the potential modulation of PA by these compounds, we constructed a “metabolite-gene-compound” network (Supplementary S4) based on Figure 5A and Figure 4C, which revealed 31 compounds with potential regulatory effects on PA. Notably, existing studies have provided insights into the roles of certain components of QXD, such as quercetin, kaempferol (+)-catechin, and stigmasterol, in modulating the content and function of PA. For instance, quercetin has been shown to enhance hepatic lipid metabolism, particularly ω-oxidation, and inhibit acetyl-CoA carboxylase (ACC), a key enzyme involved in *de novo* fatty acid synthesis, thereby leading to a significant reduction in PA formation (Gnoni et al., 2009; Hoek-van den Hil et al., 2013) (+)-Catechin has been demonstrated to reduce PA accumulation and ameliorate the ethanol-induced oxidative state of the liver (Ryle et al., 1983). Additionally, it has been found to provide protection against PA-induced lipotoxicity in rat cortical astrocytes (Wong et al., 2014). Stigmasterol, on the other hand, has been shown to inhibit fatty acid synthase (FASN), a key enzyme in the fatty acid synthesis pathway, thereby reducing PA synthesis (Monika and Geetha, 2015; Morigny et al., 2021). However, further investigations are warranted to ascertain whether these compounds present in QXD can effectively inhibit the content, function, and specific regulatory mechanisms of PA in asthmatic mice. Such studies will contribute to a more comprehensive understanding of the therapeutic potential of QXD in managing asthma.

Several limitations must be acknowledged in this study. For instance, the absence of a positive control drug in the *in vivo* experiments conducted in mice represents a notable limitation. Additionally, while numerous studies have addressed the quality control markers of the herbs contained in QXD, such as astragaloside for *A. membranaceus* (Bi. et al., 2020b), epimedoside for *E. brevicornum* (Shahnawaz, N et al., 2014), and polydatin for *P. cuspidatum* (Gao et al., 2016), the quantification of these quality control markers in QXD was found to be incomplete. Previous research conducted by Zhang et al. utilized mass spectrometry to identify compounds, including astragaloside, epimedoside, and polydatin (Zhang et al., 2020). Based on these findings, it is proposed that astragaloside, epimedoside, and polydatin could potentially serve as quality control markers for QXD. To address this limitation, future studies will utilize traditional Chinese medicine (TCM) fingerprinting and mass spectrometry techniques to detect and quantify standardized quality control markers in QXD.

## 5 Conclusion

Through the application of metabolomics combined with network pharmacological analysis, our study has unveiled the potential of QXD to reduce serum PA levels in asthmatic mice. Furthermore, our findings indicate that QXD exerts inhibitory effects on PA-induced expression and secretion of IL-6 by targeting the MAPK signaling pathway. Additionally, our *in vivo* experiments have provided evidence of QXD’s ability to decrease the number of infiltrating macrophages and IL-6 expression in lung macrophages. These results collectively suggest that the therapeutic mechanism of QXD in treating asthma may be associated with the regulation of metabolic pathways, specifically the inhibition of PA content and function. In conclusion, the integration of metabolomics and network pharmacology holds promise for elucidating the molecular mechanisms underlying traditional Chinese medicine.

## Data Availability

The datasets presented in this study can be found in online repositories. The names of the repository/repositories and accession number(s) can be found in the article/Supplementary Material.
