# Supplementary material for: Exploration of the mechanism of Qi-Xian decoction in asthmatic mice using metabolomics combined with network pharmacology
Source: Front Mol Biosci. 2023 Dec 13;10:1263962. doi: 10.3389/fmolb.2023.1263962 (PMC10753777; doi:10.3389/fmolb.2023.1263962)
Supplement: Supplementary file 1 [file DataSheet1.ZIP › Supplementary+Table/Table/Table 1 Composition of QXD.docx]

**Table 1:**

**Composition of QXD**

| **Herb** | **Latin name** | **Application (g)** |
| --- | --- | --- |
| Huangqi | Astragalus membranaceus | 30 |
| Yinyanghuo | Epimedium brevicornum | 30 |
| Bajitian | [Morinda officinalis](https://old.tcmsp-e.com/tcmspsearch.php?qr=Morindae Officinalis Radix&qsr=herb_en_name&token=c79a48bbed602f6392339d34f5c809db) | 30 |
| Huzhang | [Polygonum cuspidatum](https://old.tcmsp-e.com/tcmspsearch.php?qr=Polygoni Cuspidati Rhizoma Et Radix&qsr=herb_en_name&token=c79a48bbed602f6392339d34f5c809db) | 30 |
| Chuanxiong | Ligusticum [chuanxiong](https://old.tcmsp-e.com/tcmspsearch.php?qr=Chuanxiong Rhizoma&qsr=herb_en_name&token=c79a48bbed602f6392339d34f5c809db) | 15 |
| Dihuang | [Rehmannia g](https://old.tcmsp-e.com/tcmspsearch.php?qr=Rehmanniae Radix Praeparata&qsr=herb_en_name&token=c79a48bbed602f6392339d34f5c809db)lutinosa | 30 |
| Pipaye | [Eriobotrya j](https://old.tcmsp-e.com/tcmspsearch.php?qr=Eriobotryae Folium&qsr=herb_en_name&token=c79a48bbed602f6392339d34f5c809db)aponica | 30 |
| Xuanfuhua | [Inula j](https://old.tcmsp-e.com/tcmspsearch.php?qr=Inulae Flos&qsr=herb_en_name&token=c79a48bbed602f6392339d34f5c809db)aponica | 9 |
