# Supplementary material for: Exploration of the mechanism of Qi-Xian decoction in asthmatic mice using metabolomics combined with network pharmacology
Source: Front Mol Biosci. 2023 Dec 13;10:1263962. doi: 10.3389/fmolb.2023.1263962 (PMC10753777; doi:10.3389/fmolb.2023.1263962)
Supplement: Supplementary file 1 [file DataSheet1.ZIP › Supplementary+Table/Table/Table 2 Sequences of Primers.docx]

**Table 2**

**Sequences of Primers.**

| **Primers** | **Sequence (5'to3')** |
| --- | --- |
| MUC5AC (F) | AATGGCGAGTCTGTGCAGGA |
| MUC5AC (R) | CACCAGGTGTGGCATTGTGA |
| IL-6 (F) | TCTATACCACTTCACAAGTCGGA |
| IL-6 (R) | GAATTGCCATTGCACAACTCTTT |
| β-actin (F) | GGCTGTATTCCCCTCCATCG |
| β-actin(R) | CCAGTTGGTAACAATGCCATGT |
