# Supplementary material for: Exploration of the mechanism of Qi-Xian decoction in asthmatic mice using metabolomics combined with network pharmacology
Source: Front Mol Biosci. 2023 Dec 13;10:1263962. doi: 10.3389/fmolb.2023.1263962 (PMC10753777; doi:10.3389/fmolb.2023.1263962)
Supplement: Supplementary file 1 [file DataSheet1.ZIP › Supplementary+Table/Table/Table 3 Differential metabolic signature after QXD treatment of asthma.docx]

**Table 3**

**Differential metabolic signature after QXD treatment of asthma.**

| **NO** | **Metabolites** | **Formula** | **M/Z** | **RT(min)** | **VIP** | **Log2**  **(A/N)** | **Log2**  **(AQH/A)** |
| --- | --- | --- | --- | --- | --- | --- | --- |
| 1 | 7(14)-Bisabolene-2,3,10,11-tetrol | C15H28O4 | 317.1973406 | 5.691566667 | 0.30 | 3.54↑** | -1.19↓* |
| 2 | Callystatin A | C29H44O4 | 501.3229614 | 9.04485 | 1.10 | 3.27↑** | -2.21↓* |
| 3 | Citalopram (propionic acid derivative) | C19H16FNO3 | 649.2146311 | 5.426183333 | 0.53 | 3.11↑** | -0.95↓* |
| 4 | Palmitic acid | C16H32O2 | 301.2388799 | 9.61905 | 0.36 | 3.08↑* | -1.83↓* |
| 5 | Uzarigenin 3-[xylosyl-(1->2)-rhamnoside] | C34H52O12 | 670.379459 | 7.641516667 | 0.72 | 2.90↑** | -0.82↓* |
| 6 | Methyl (R)-9-hydroxy-10-undecene-5,7-diynoate glucoside | C18H24O8 | 413.1458462 | 4.9061 | 0.38 | 2.89↑** | -0.89↓* |
| 7 | Pisumionoside | C19H32O9 | 403.1977175 | 4.765166667 | 0.42 | 2.60↑** | -3.60↓** |
| 8 | Cinncassiol C | C20H28O7 | 425.1823674 | 7.638033333 | 0.52 | 2.49↑** | -1.43↓** |
| 9 | RHODOMYRTOXIN B | C24H28O7 | 473.1822158 | 6.938133333 | 0.55 | 2.42↑** | -1.01↓** |
| 10 | (2E,6E)-1-Hydroxy-2,6,10-farnesatrien-9-one | C15H24O2 | 471.3485522 | 11.44598333 | 0.83 | 2.29↑** | -2.08↓** |
| 11 | MG(0:0/14:1(9Z)/0:0) | C17H32O4 | 345.2286739 | 6.818266667 | 0.45 | 2.20↑** | -0.88↓* |
| 12 | 17-phenyl trinor PGF2α isopropyl ester | C26H38O5 | 475.2683473 | 7.916783333 | 0.60 | 2.18↑** | -1.95↓* |
| 13 | (±)-1,4-Nonanediol diacetate | C13H24O4 | 289.166026 | 5.013016667 | 0.35 | 2.16↑** | -1.02↓* |
| 14 | QX-314 | C16H26N2O | 307.2031347 | 9.206716667 | 0.47 | 1.97↑** | -0.89↓* |
| 15 | 3alpha,4beta,7alpha-Trihydroxy-5beta-cholan-24-oic Acid | C24H40O5 | 453.2864381 | 7.916783333 | 1.46 | 1.95↑** | -1.46↓* |
| 16 | Pantetheine | C11H22N2O4S | 279.1376638 | 4.5677 | 0.19 | -4.49↓* | 5.54↑* |
| 17 | Lubiminol | C15H26O3 | 299.1867329 | 9.935016667 | 0.34 | -3.58↓* | 4.60↑** |
| 18 | 20-Oxo-leukotriene E4 | C23H35NO6S | 452.2101721 | 7.251733333 | 0.53 | -2.12↓** | 2.26↑* |
| 19 | 2-Naphthalenesulfonic acid | C10H8O3S | 189.0021432 | 4.574983333 | 1.02 | -2.08↓* | 2.16↑* |
| 20 | N-(3-oxo-dodecanoyl)-homoserine thiolactone | C16H27NO3S | 358.1699534 | 8.896166667 | 0.68 | -1.87↓** | 2.22↑* |
| 21 | Spermidine | C7H19N3 | 146.1651725 | 0.566383333 | 0.35 | -1.82↓* | 1.24↑* |
| 22 | DOPA sulfate | C9H11NO7S | 322.0247113 | 4.5254 | 0.25 | -1.62↓** | 0.99↑* |
| 23 | 17-dimethylarsinoyl-9Z-heptadecenoic acid | C19H37AsO3 | 387.1889931 | 3.607783333 | 0.15 | -1.57↓** | 1.27↑** |
| 24 | N-(3-TRIFLUOROMETHYLPHENYL)PIPERAZINE (TFMPP) | C11H13F3N2 | 459.2006528 | 7.251733333 | 0.49 | -1.44↓* | 1.35↑* |
| 25 | Meptazinol glucuronide | C21H31NO7 | 408.2032591 | 7.2661 | 1.38 | -1.39↓* | 1.39↑* |
| 26 | 2-(2H-1,3-benzodioxol-5-yl)-2-oxoacetic acid | C9H6O5 | 232.9854875 | 4.5677 | 0.95 | -1.38↓* | 1.83↑* |
| 27 | 4-Chloro-17alpha-methyl-17beta-hydroxy-4-androsten-3-one | C20H29ClO2 | 381.1842397 | 7.251733333 | 0.88 | -1.34↓* | 1.23↑* |
| 28 | Trifluridine | C10H11F3N2O5 | 277.0442351 | 4.5254 | 0.48 | -1.25↓* | 0.67↑* |
| 29 | N-Acetylaspartylglutamic acid | C11H16N2O8 | 303.0836921 | 1.1402 | 0.60 | -1.12↓** | 0.64↑** |
| 30 | DEQUALINIUM | C30H38N4 | 472.3427942 | 10.25435 | 0.58 | -1.09↓** | 0.70↑* |

↑ indicates increase; ↓ indicates decrease. **P* < 0.05; * **P* < 0.01.
