# Supplementary material for: Exploration of the mechanism of Qi-Xian decoction in asthmatic mice using metabolomics combined with network pharmacology
Source: Front Mol Biosci. 2023 Dec 13;10:1263962. doi: 10.3389/fmolb.2023.1263962 (PMC10753777; doi:10.3389/fmolb.2023.1263962)
Supplement: Supplementary file 1 [file DataSheet1.ZIP › Supplementary+Table/Table/Table 4 Gene associated with both asthma and QXD.docx]

**Table 4**

| **Gene Symbol（126 genes）** |
| --- |
| ADRB2,CHRM3,PTGS1,PTGS2,HSP90AA1,CHRM1,ADRA1A,CHRM2,ADRA1B,SLC6A4,OPRM1,BCL2,JUN,CASP3,CASP8,PRKCA,PON1,ESR1,DPP4,NOS2,PPARG,KDR,MAPK14,NR3C1,ADRA2A,SLC6A2,AKR1B1,PLAU,LTA4H,MAOA,ADRB1,HTR3A,IL4,MAPK8,MMP1,STAT1,HMOX1,CYP3A4,CYP1A1,ICAM1,SELE,VCAM1,ALOX5,GSTP1,PSMD3,GSTM1,SLPI,MMP3,EGFR,VEGFA,CCND1,MMP2,MMP9,MAPK1,EGF,IL6,NFKBIA,SOD1,ERBB2,CAV1,F3,IL1B,CCL2,PTGER3,NOS3,IL2,THBD,SERPINE1,COL1A1,IFNGR1,IL1A,MPO,NFE2L2,NQO1,PARP1,CRP,CXCL10,CHUK,SPP1,IGFBP3,CD40LG,IRF1,CA2,ADRA2B,CAT,PCNA,GSTA1,IRF3,STAT3,FGFR1,FLT1,PDGFB,MAPK3,PDGFRB,IVL,COMT,TIMP1,EDNRA,CD4,FGF2,FAS,FCER1A,TLR4,SP1,G6PD,CREB1,CDH1,MUC5AC,HRH1,DRD2,ACTA2,BTK,CSF2,IGHG1,F7,F9,MYC,NCOA1,NCOA2,PRKCD,PRKCE,PRSS1,PTPN2,TNF,TP53,PIK3CG |

**Gene associated with both asthma and QXD.**
