# Supplementary figures and images for: Exploration of the mechanism of Qi-Xian decoction in asthmatic mice using metabolomics combined with network pharmacology
Source: Front Mol Biosci. 2023 Dec 13;10:1263962. doi: 10.3389/fmolb.2023.1263962 (PMC10753777; doi:10.3389/fmolb.2023.1263962)

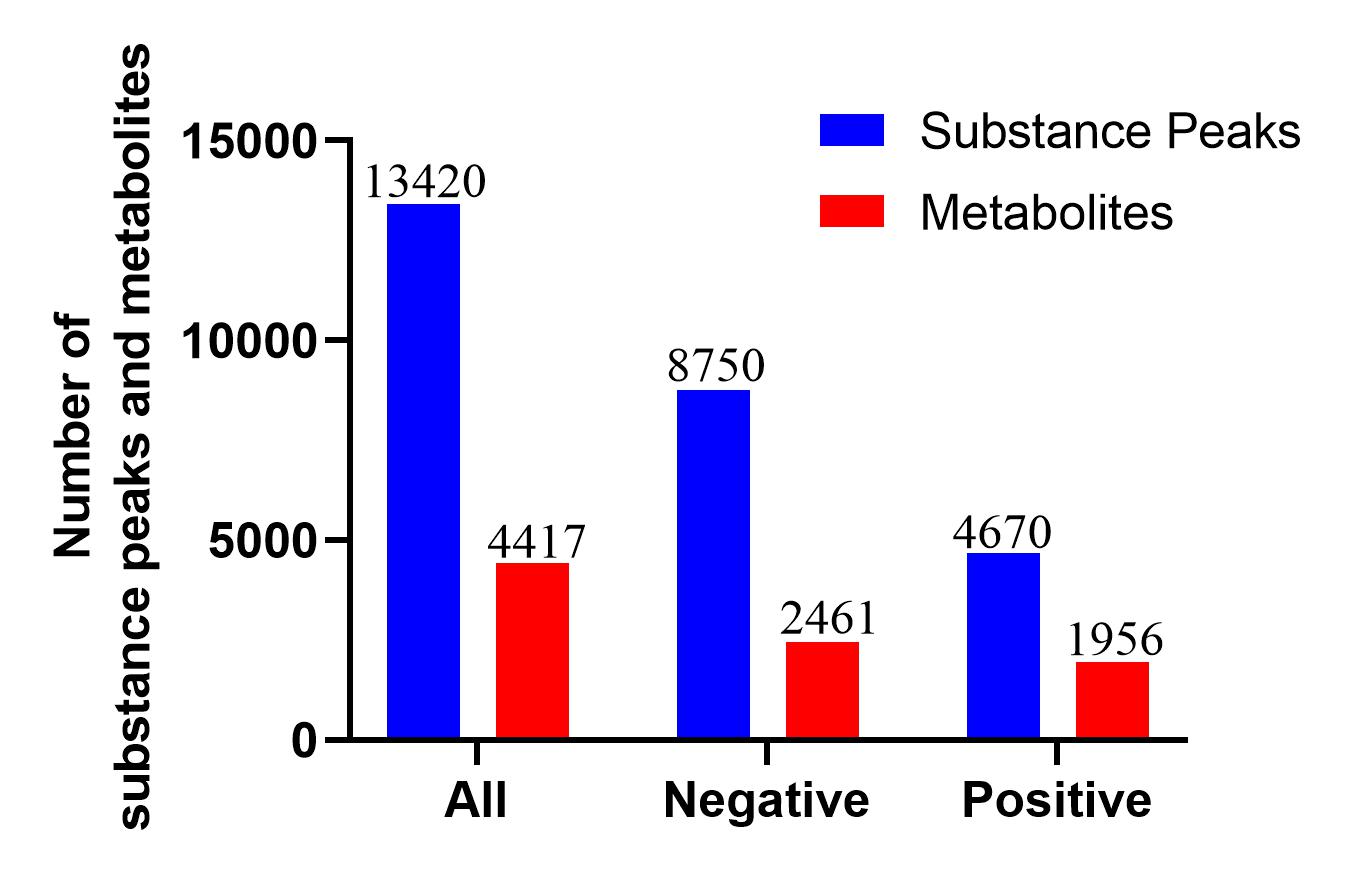

Supplement: Supplementary file 1 [file DataSheet1.ZIP › Supplementary+Table/1. Supplementary S1.jpg]

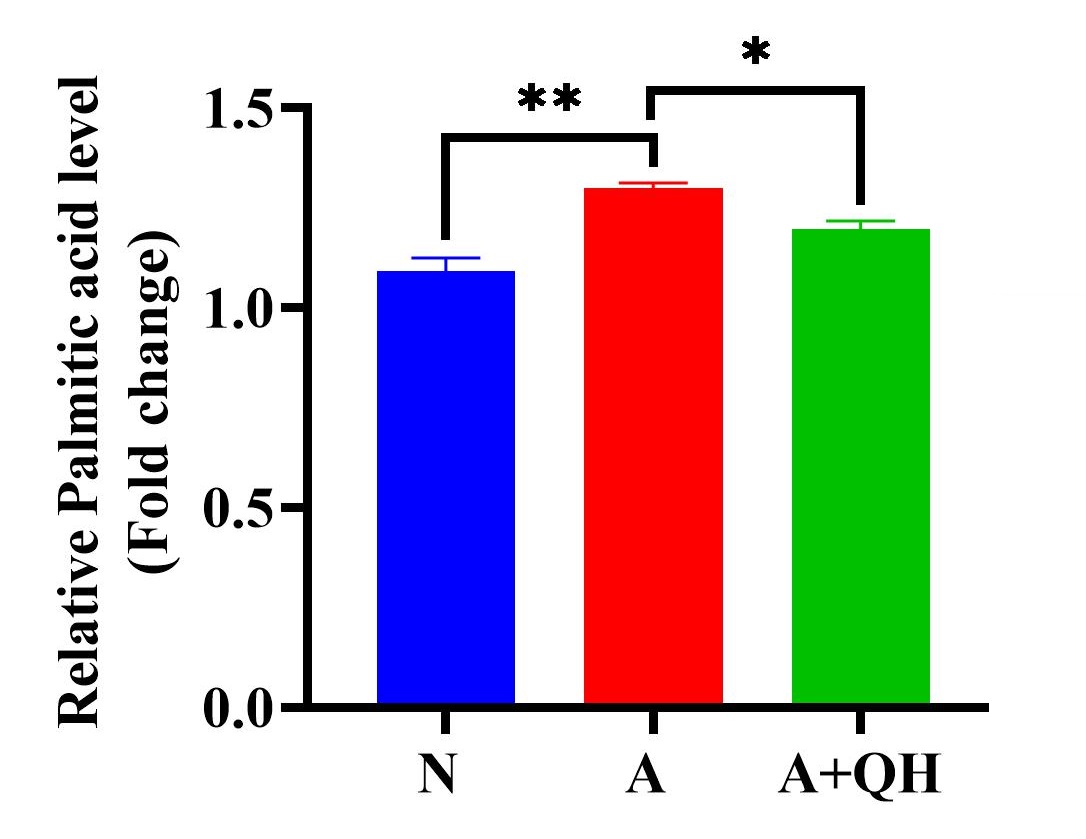

Supplement: Supplementary file 1 [file DataSheet1.ZIP › Supplementary+Table/5. Supplementary S5.jpg]
